# Dietary Flaxseed in Non-Small Cell Lung Cancer Patients Receiving Chemoradiation

**DOI:** 10.4172/2161-105X.1000154

**Published:** 2013-08-30

**Authors:** Abigail T Berman, Jason Turowski, Rosemarie Mick, Keith Cengel, Nicole Farnese, Lisa Basel-Brown, Clementina Mesaros, Ian Blair, James Lawson, Melpo Christofidou-Solomidou, James Lee, Ramesh Rengan

**Affiliations:** 1 Department of Radiation Oncology, University of Pennsylvania, Philadelphia, USA; 2 Department of Medicine, Pulmonary Allergy and Critical Care Division, University of Pennsylvania, Philadelphia, USA; 3 Department of Biostatistics and Epidemiology, University of Pennsylvania, Philadelphia, USA; 4 Department of Pharmacology, University of Pennsylvania, Philadelphia, USA

**Keywords:** Flaxseed, Lignan, Radiation, Isoprostane, 8-oxo dGuo, Non-small cell lung cancer, Radiation induced lung injury, RILI, Pneumonitis, Fibrosis, Esophagitis

## Abstract

**Purpose:**

The standard of care in Locally-Advanced Non-Small Cell Lung Cancer (LA-NSCLC) is chemotherapy and radiation; however, Radiation-Induced Lung Injury (RILI), which may be prevented by the anti-inflammatory and anti-oxidant properties of Flaxseed (FS), impedes its maximum benefit.

**Materials and Methods:**

Patients with LA-NSCLC requiring definitive RT were randomized to one FS or control muffin daily from start to 2 weeks after RT. Blood and urine were collected to quantify plasma FS metabolites, Enterodione (ED) and Enterolactone (EL), and urinary oxidative stress biomarkers, 8, 12-iso-iPF2a-VI (isoprostane) and 8-oxo-7,8-dihydro-2′deoxyguanosine (8-oxo-dGuo). Tolerability was defined as consuming ≥ 75% of the intended muffins and no ≥ grade 3 gastrointestinal toxicities.

**Results:**

Fourteen patients (control,7; FS,7) were enrolled. The tolerability rates were 42.9 versus 71.4% (p=0.59) for FS and control, respectively. Mean percentages of intended number of muffins consumed were 37% versus 73% (p=0.12). ED and EL increased at onset of FS and decreased with discontinuation, confirming bioavailability. Isoprostane and 8-oxo-dGuo were detectable. There was a trend towards decreased rates of pneumonitis in FS.

**Conclusions:**

This is the first study to report FS bioavailability and quantify oxidative stress markers in NSCLC patients. FS in the administered muffin formulation did not meet tolerability criteria. Given the promising mechanism of FS as a radioprotectant, further investigations should focus on the optimal method for administration of FS.

## Introduction

The standard of care for patients with unresectable, locally-advanced Non-Small Cell Lung Cancer (NSCLC) is definitive Radiation Therapy (RT) given with or without chemotherapy. However, outcomes with this modality are quite poor with 5-year survival estimates at 16% [1]. A major barrier to improved outcomes is the development of Radiation-Induced Lung Injury (RILI). RILI occurs in 30% or greater of lung cancer patients [2] and manifests on a spectrum from acute radiation pneumonitis, an inflammatory state occurring within weeks to 6 months of RT, to chronic radiation fibrosis, characterized by replacement of functional lung parenchyma with fibrotic tissue usually occurring months to years following therapy. Recent studies have shown that radiation causes a state of oxidative stress characterized by increased generation of free radicals. Volatile reactive oxygen (ROS) and nitrogen (RNS) species have been shown *in vivo* and *in vitro* to accelerate lipid peroxidation, oxidize DNA and cellular proteins, as well as activate pro-inflammatory cytokines. Reducing oxidative stress would hypothetically prevent RILI and improve the therapeutic index of radiotherapy in lung cancer. No known effective pharmacologic therapy to prevent this challenging complication of radiotherapy exists.

In this study, two urinary biomarkers of systemic oxidative stress, 8, 12-iso-iPF2a-VI isoprostane and 8-oxo-7, 8-dihydro-2′deoxyguanosine (8-oxo-dGuo), were investigated. Isoprostanes, oxidative stress biomarkers, are prostaglandin-like compounds formed by the peroxidation of arachidonic acid by free radicals. Reactive oxygen species (ROS) cause the 8-hydroxylation of guanine (8-oxo-Guo) [3]. This molecule and its deoxyribonucleoside counterpart (8-oxo-dGuo) are excreted in the urine ^4^ and the rate of urinary excretion of 8-oxo-dGuo parallels the rate of oxidative stress. Biochemical studies have revealed that certain populations (smokers, lung cancer, COPD) have baseline elevations in 8-oxo-dGuo levels that do not trend back to epidemiological baseline levels after an oxidative insult was terminated [4]. Flaxseed (FS) is a non-toxic, dietary wholegrain composed of omega-3 fatty acids and a lignan complex (FLC) with potent anti-inflammatory, antioxidant and anti-fibrotic properties. FS is the richest known source of the mammalian lignan precursor, Secoisolariciresinol Diglucoside (SDG), which is metabolized via enteric bacteria to Enterodione (ED) and Enterolactone (EL). As a plant phenolic, SDG was shown *in vitro* to have direct hydroxyl radical scavenging properties, limit the respiratory burst of White Blood Cells (WBCs) and to inhibit lipid peroxidation [5]. Flaxseed has been reported to provide health benefits in several disease conditions including hypercholesterolemia, diabetes, menopause [6], cardiovascular health, metabolic syndrome and bone mineral metabolism. Extensive research by our group and elsewhere [7–10], has helped characterize the benefits of the wholegrain in numerous clinical scenarios. This was our first clinical application of wholegrain FS to mitigate the effects of thoracic radiation. Our long-term goal is to improve clinical responses and therapeutic indices of RT for thoracic malignancies while providing adequate radioprotection against the side effects on normal lung parenchyma.

The primary objective of this study was to ascertain the feasibility of incorporating a validated radioprotector, Flaxseed (FS), in the treatment regimen of patients with advanced NSCLC receiving chemotherapy and thoracic radiation. We specifically focused on whether flaxseed muffins would have a reasonable toxicity and tolerability index for patients receiving chemoradiation. We additionally sought to validate FS bioavailability by quantifying serum markers of metabolism and quantifying urinary markers of systemic oxidative stress before, during, and after RT in the presence and absence of concurrent FS supplementation. An additional component of the study aimed to establish if serum lignan levels and/or urinary markers of systemic oxidative stress demonstrated a dose-response relationship with 20 versus 40 grams of flaxseed.

## Materials and Methods

This randomized, double-blinded, placebo-controlled study was conducted in accordance with the IRB. Eligible patients were 18 years or older and had a diagnosis of non-small cell lung cancer (NSCLC) requiring definitive thoracic and mediastinal radiation. The study was amended in February 2009 to require concurrent chemotherapy with RT. Patients with metastatic disease were eligible.

### Primary and secondary outcomes

Primary outcomes were toxicity and tolerability of dietary flaxseed administration during chemoradiation. Secondary endpoints included urinary biomarkers of systemic oxidative stress and serum levels of flaxseed metabolites enterodione and enterolactone.

### Enrollment criteria

The following were criteria for ineligibility: prior thoracic RT; Gastrointestinal (GI), liver, or kidney disease that could result in altered metabolism or excretion of the study medication were excluded, including major Gastrointestinal (GI) surgery, ulcerative colitis, regional enteritis, or GI bleeding; administration of an investigational drug, amifostine or Mucomyst (N-acetyl-cysteine) within 14 days; known hypersensitivity to flaxseed (or its metabolites) or wheat, or those who were currently taking flaxseed, flax-containing products, soybeans, soy-containing products, or other herbals or botanicals. Those taking or had taken Vitamin E in excess of 30 IU within the prior 21 days were excluded.

### Experimental design

**Figure 1:**
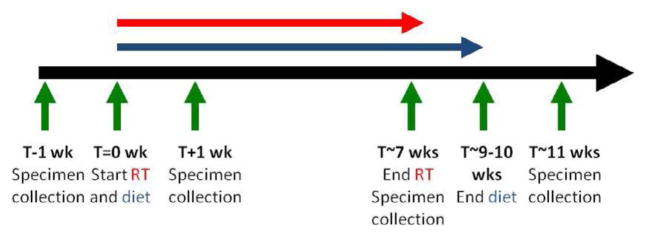
Experimental Plan. Patients were randomized to flaxseed or control diet initiated at the start of RT, given until 2 weeks after end of RT. Specimen (blood and urine collections) performed at pre-RT, week 1, end RT, and 1 month post-RT time points.

We elected to perform this trial as a randomized placebo-controlled trial because the outcome of tolerability is subjective and it would be best to have a control arm, thus minimizing bias. The trial randomized patients to two dose levels. A standard 1:1 randomization method was applied in this trial, the randomization was performed by the General Clinical Research Center, and therefore the investigators and patients were blinded to the randomization. Twelve patients were first randomized to consume one muffin per day (20 g of flaxseed, 6 patients) or one Durham wheat muffin (control) per day (6 patients) starting on the day their definitive RT begins and for the duration of their therapy definitive RT until two weeks after completion of RT (9–10 weeks total). If 2 subjects on the first dose level failed to tolerate the diet, then the trial would be suspended and the randomization code broken. If tolerated, then an additional 12 patients were intended to be randomized to a second dose level of two muffins per day (40 g). Figure 1 shows the experimental design of this study. Patients and investigators were blinded as to which type of muffin each subject was assigned. Ground wholegrain flaxseed (Dr. Jack Carter, University of North Dakota) was baked into muffins according to a recipe developed by the metabolic kitchen of the Clinical Translational Research Center (CTRC). The muffins were isocaloric and were prepared as described by Bloedon et al. [11]. Blood and urine samples were obtained at four time points (Figure 1): 1) Baseline – one week prior to starting RT and FS diet; 2) End of week 1 of RT and FS diet; 3) End of RT with FS diet still ongoing; 4) One month after end of RT (2 weeks after end of FS diet). Subjects were asked to eat the muffin(s) at least 4 hours before sample collection. Plasma was analyzed for metabolites ED and EL of flaxseed lignan. Urine was analyzed for isoprostane and 8-oxo-7, 8-dihydro-2′-deoxyguanosine (8-oxo-dGuo) levels each time. Plasma and urine collection was incomplete in 8 patients due to withdrawal from study due to progressive disease (2) or tolerability (4), or lost to follow up due to patient (1) or hospitalization (1).

### Radiation treatment planning

All patients underwent CT-based treatment planning for RT. Gross Tumor Volume (GTV) was defined as all known sites of disease including pathologically enlarged (≥ 1 cm in short axis) or FDG-avid lymph nodes. Elective nodal radiation was not permitted. Total dose to gross disease was 60–80 Gray (Gy) administered in 2 Gy fractions given daily 5 days per week. The Clinical Target Volume (CTV) was created from the GTV by adding a 1 cm margin. In 11 out of 14 patients, 4D-CT and PET/CT fusion was used for treatment planning. Dose constraints were as follows: volume of lung receiving 20Gy (V20) less than 35%, maximum spinal cord doss less than 45Gy, dose to 50% of cardiac volume (D50) less than 40Gy. The esophagus dose was calculated for each treatment plan but was not a dose-limiting structure.

### Quantifying flaxseed lignan metabolites

Circulating plasma levels of ED and EL at the four specimen time points were determined by liquid chromatogram phys tandem mass spectrometry (LC/MS/MS) as described earlier [12,9] using commercially available standards in 95% purity (Chromadex, Inc., Santa Ana, CA). Urinary 8,12-*iso*-iPF2a-VI [13] and 8-oxo-7,8-dihydro-2′-deoxy-guanosine (8-oxo-dGuo) levels [14] were measured and normalized to urine creatinine level 8-oxo-dGuo levels were measured by high-performance liquid chromatography-electrospray tandem mass spectrometry [15].

### Patient tolerability/toxicity logs

Patients were asked to log how many muffins were consumed during the study. Toxicity, including pneumonitis, was scored according to CTCAE version 3.0. Tolerability was defined as the ability consume ≥ 75% of the intended number of muffins and the lack of dose-limiting toxicity, defined as ≥ grade 3 gastrointestinal toxicity. The desired tolerability rate was 80%.

### Statistical methods

Fisher’s exact test was used to calculate whether there was a statistically significant difference between the tolerability and rates of pneumonitis and esophagitis. The Wilcoxon rank sum test was used to calculate whether there was a statistically significant difference between the percent of prescribed muffins ingested in the two arms.

## Results

### Patient accrual and compliance

Fourteen patients were enrolled in this study and consumed at least one control (n=7) or one flaxseed muffin (n=7). Table 1 shows patient and tumor characteristics. Five patients in the control group and six in the FS group had stage III NSCLC; the others had oligometastatic (stage IV) disease, for whom definitive thoracic radiotherapy was recommended. The median radiation dose was 7200 and 7000 cGy in the control and flaxseed groups, respectively. One patient randomized in the FS cohort was found to have metastatic disease at time of treatment, resulting in early termination of thoracic radiation. A total of four patients (1 control, 3 FS) had concurrent chemotherapy with another 8 patients receiving sequential chemotherapy. Two patients did not receive any chemotherapy.

Table 2A shows subject compliance and muffin ingestion. Tolerability rate was five of seven (71.4%) patients from the control group and three of seven (42.9%, p=0.59) from the flaxseed group. Of the control group, mean 73% (range 8.5–100%) of the muffins were consumed and of the flaxseed group, 37% (range 2.8–100%, p=0.12) of the muffins were consumed. Reasons cited for intolerability were anorexia, odynophagia and flaxseed formulation. The trial did not proceed to the second dose level as, in the first dose level, the requirement that patients be able to consume ≥ 75% of the intended number of muffins was not met. Blood and urine were collected from six patients prior to start of radiation and four patients after RT.

### Detection of plasma lignan levels

**Figure 2:**
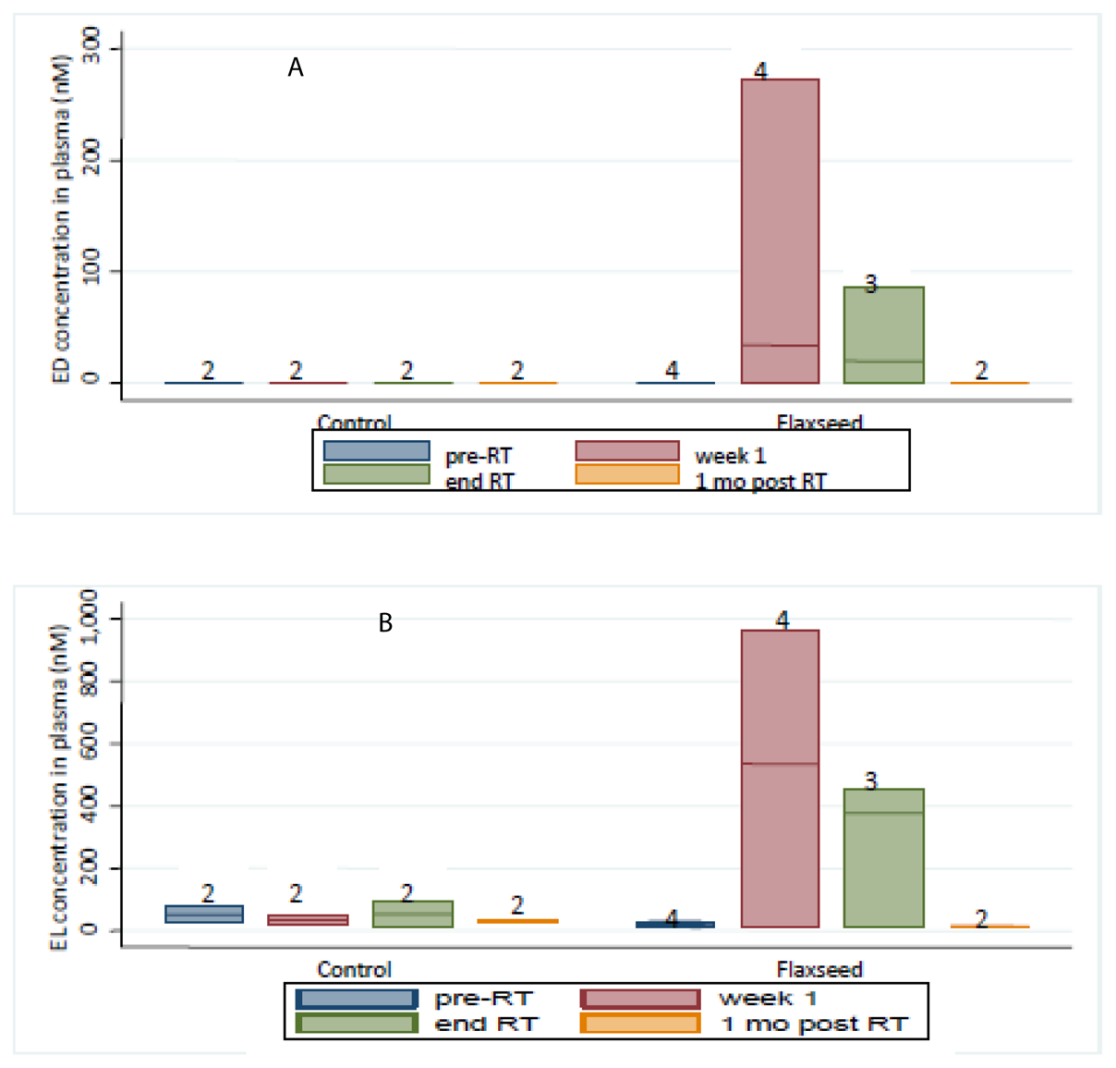
Detection of flaxseed lignan metabolites in blood. Circulating lignan (A showing ED and B showing EL) levels in plasma of patients at four time points (pre-RT, week 1, end RT, 1 month post-RT) were determined using GC/MS/MS. Data is represented by a box plot

Figure 2 shows all collection times throughout the study. Plasma ED levels for patients not on flaxseed was undetectable; plasma EL levels in control patients also were very low. Both plots show expected elevations in plasma ED and EL levels at onset of flaxseed administration and a drop-off of ED and EL levels when FS supplementation was discontinued. There were three patients in the flaxseed cohort who were prescribed antibiotics, levofloxacin (fluoroquinolone) and metronidazole (nitroimidazole) and these patients had detectable ED and EL levels.

### Effect of dietary flaxseed on urinary markers of systemic oxidative stress

**Figure 3:**
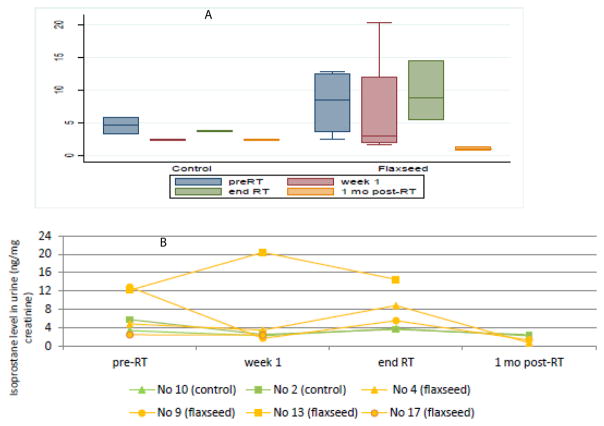
Detection of isoprostane levels in urine. Urine was measured for 8,12, F2 isoprostane levels and normalized to creatinine level (ng/mg creatinine). Panel A shows a box plot of isoprostane levels in both diet groups (control and flaxseed) at the four time points. Panel B demonstrates the isoprostane level trend in 6 patients (2 control, 4 flaxseed).

**Figure 4:**
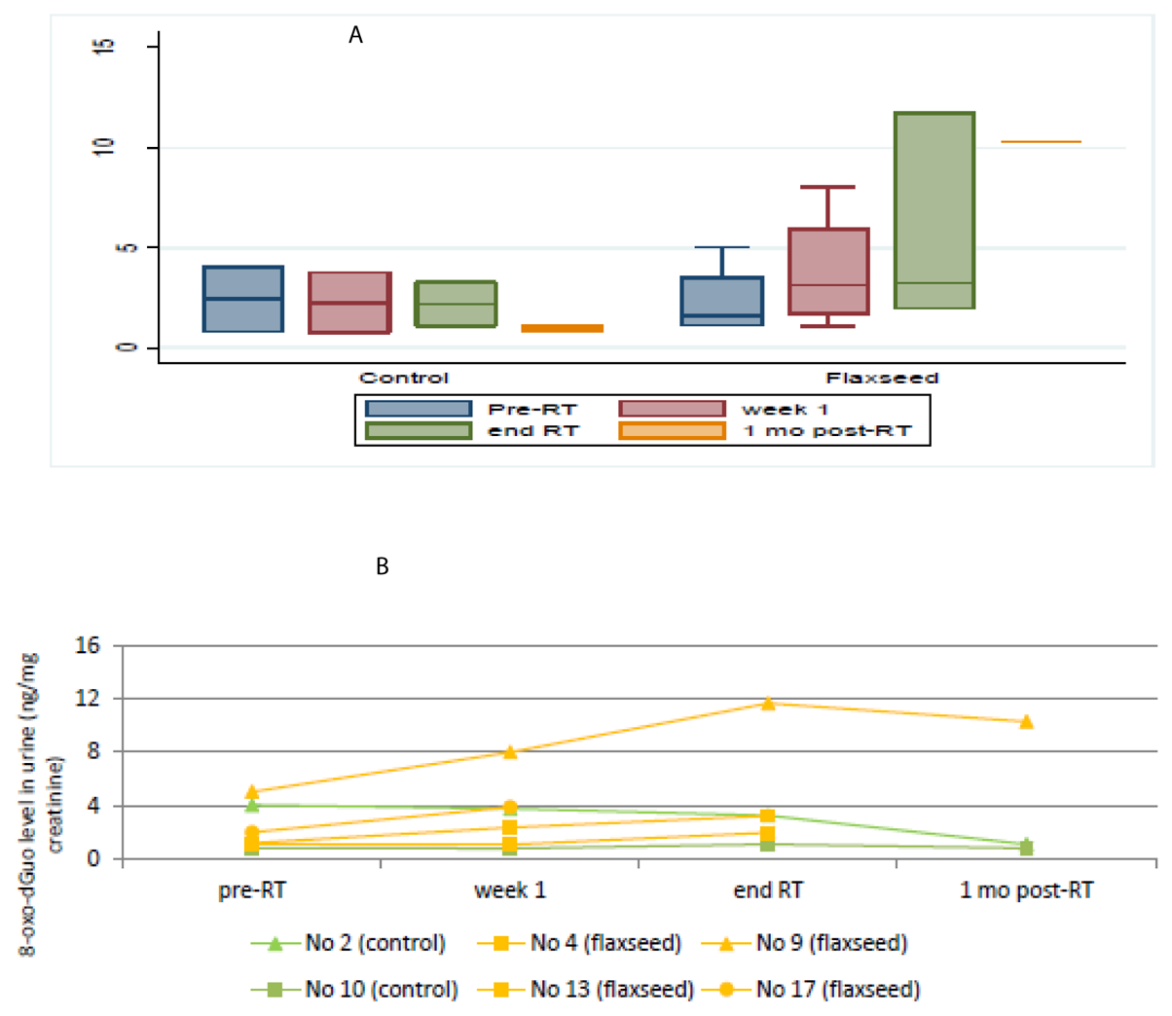
Detection of 8-oxo-dGuo levels in urine. Urine was measured for levels of 8 8-oxo-7,8-dihydro-2′-deoxyguanosine and normalized to creatinine level (ng/mg creatinine). Panel A shows a box plot 8-oxo-dGuo levels in both diet groups (control and flaxseed) at the four time points. Panel B shows the urinary 8-oxo-dGuo level trend in 6 patients (2 control, 4 flaxseed).

Systemic oxidative stress can be estimated via urinary isoprostane measurements, normalized to urine creatinine. Panel A of Figure 3 shows that mean isoprostane levels decrease at 1 month after RT. There were six patients (2 non-FS, 4 FS) whose blood and urinalyses were obtained at 1 month post-RT; these individuals are graphed in Figure 3B. All patients displayed elevated isoprostane levels after completion of radiotherapy. Urinary 8-oxo-dGuo levels were also measured and normalized to creatinine as a marker of intracellular oxidative stress (Figure 4, Panels A–B). Similarly, 8-oxo-dGuo decreased one month after completion of radiotherapy.

### Adverse effects and clinical outcomes

There were no adverse effects directly related to administration of flaxseed-containing muffins. Table 2B displays rates of esophagitis (odynophagia, dysphagia, gastrointestinal reflux symptoms, and possibly nausea and vomiting) and pneumonitis in each diet group exposed to thoracic radiation. There were four cases of esophagitis in the flaxseed arm compared to six in the control arm (*p*=0.56 by Fisher’s exact test). There were no cases of pneumonitis in the flaxseed arm compared to three in the control arm (*p*=0.19).

The median follow up was 9.9 months. Six of 7 (86%) patients died in each arm. Two-year overall survival was found to be 30% in both arms. Of the patients who died in the control arm, three patients had Distant Metastases (DM), two of whom had metastases on presentation as well, and one had a Local Recurrence (LR). In the flaxseed arm, there were three patients with DM (1 pre-existing DM) and 1 LR.

## Discussion

We assessed the feasibility of incorporating flaxseed in the treatment of a small cohort of patients with advanced non-small cell lung cancer. Adherence to the flaxseed diet in the baked muffin formulation was below required levels for the study and therefore the trial was terminated after the first dose level. Here, we present the initial analysis of the patients enrolled. Markers of flaxseed metabolism, enterodione and enterolactone, were detected in the sera of the flaxseed group undergoing therapy. Urinary biomarkers of systemic oxidative stress (isoprostane and 8-oxo-D-guo) increased in response to radiotherapy. Trends toward reduced rates of pneumonitis were seen in FS-fed patients. More patients in the FS arm received concurrent chemotherapy than in the control arm; and despite this difference, pneumonitis rates were lower in the FS arm.

Poor adherence to flaxseed supplementation in the muffin formulation was likely multi factorial. Patients noted intolerable taste and/or texture with flaxseed muffins, which is of prime importance in patients with anorexia and odynophagia due to chemoradiotherapy. Recently, novel formulations of flaxseed and routes of administration have been designed and may circumvent this issue such as employing ground whole grain flaxseed as a condiment, flavor enhancer, or thickening agent.

The antioxidant mechanism of flaxseed is either by direct hydroxyl radical scavenging or by counteracting lipid peroxidation. Thompson et al. [16] in their studies of healthy volunteers ingesting varying concentrations and formulations of flaxseed [16] demonstrated the time-dependent concentration differences of ED and EL in the serum and urine of healthy patients. Our study has confirmed that ED and EL plasma concentration were quantifiable in the flaxseed group with the ED and EL levels at or greater than seen previously. While FS metabolites generally reach a plateau in the plasma within 3 weeks of supplementation in our murine models [17], the drop in levels shown here may be secondary to poor adherence to the diet or other biochemical factors.

Prior work by our group (21) had shown dietary FS given before and after thoracic radiotherapy prevented radiation induced pneumonopathy in a murine model. Lungs were evaluated at 24 hours post-RT for acute markers of radiation-induced injury and the FS-fed mice demonstrated reduced expression of lung injury biomarkers (Bax, p21, and TGF-beta1). At three weeks, there was decreased cellular injury from RT as measured by malondialdehyde (MDA) levels. At four months, mice had decreased hydroxyproline content and fewer inflammatory cells in their bronchoalveolar fluid analyses indicating less late damage.

Here we demonstrated a response in two urinary markers of systemic oxidative stress, isoprostane and and 8-oxo-dGuo. Biomarker levels increased from radiotherapy and ultimately decreased at one-month post treatment. This observation was consistent with the understanding that inflammation increases during the course of radiotherapy. As acute effects of radiation diminish and tumor size decreases, there are lower oxidative stress levels. We saw that there was an increase in 8-oxo-dGuo that did not normalize after cessation of radiotherapy. This could relate to ongoing DNA damage in tumor and normal cells persisting post-RT or in conjunction with chemotherapy or other antibiotic therapy. Isoprostane levels appeared to increase more in the flaxseed arm between week 1 and the end of RT; however, there was also a greater decrease between the ends of RT compared to week 1. Taken together, there was an overall decrease in isoprostane level from week 1 to the one month after RT. A dramatic decrease in isoprostane levels from baseline (one week prior to RT and FS treatment) occurred with initiation of flaxseed muffins. this study As seen in Figure 3, the isoprostane curve mirrors the oxidative stress curve, with baseline elevated biomarkers representing respiratory impairment and advanced cancer, preliminary drop reflecting the start of flaxseed in the presence of radiotherapy, midway spike showing the effect of radiotherapy, and, lastly, a decrease mirroring the discontinuation of all therapy. Antibiotic use can affect the bioavailability of flaxseed. In this study, patients were placed on levofloxacin, a fluoroquinolone, shown in studies to elevate markers of inflammation (isoprostanes) in otherwise healthy volunteers [17]. It is conceivable that patients on antibiotics affected their enteric flora and thus lacked an integral part of flaxseed digestion [16]. The complex interplay of chemotherapy, radiotherapy, and antibiotic therapy makes it challenging to discern what part the fluoroquinolone had in addition to chemoradiation.

Given that this is a pilot study and therefore would only have adequate power to identify large differences between groups, no definitive conclusions can be made regarding the difference in oxidative stress levels between the flaxseed and control groups. There were no cases of pneumonitis in the flaxseed arm, which was encouraging. In addition, there were fewer cases of radiation induced esophagitis. Again, these were very important findings but need to be assessed in a randomized study with adequate power. In addition, as previously noted, chemoradiation is a large cause of gastrointestinal toxicity and therefore it is unclear how much of the intolerability of the flaxseed muffins may be from the chemoradiation itself. In addition, it should be noted that there appears to be an imbalance in the type of chemotherapy received by each arm of the study. However, due to the small number of patients, we are not able to statistically evaluate for this difference.

Interpretation of these urinary biomarkers normalized to creatinine may be a source of error because of variable creatinine. With larger sample sizes, the effects of 8-oxo-dGuo and creatinine could be assessed separately in multivariable regression models [18].

Studies by our group *in vivo* [9] have revealed that dietary FS given prior to and post-thoracic radiation did not abrogate the tumoricidal properties of radiotherapy. We have also shown that dietary FS given after RT improved survival, decreased pulmonary inflammation and eventual fibrosis [10]. Additional studies are currently under way by our group to further characterize the radiosensitizing aspect of the flaxseed lignan complex as well as the dose-modifying factor of SDG, in an attempt to further improve therapeutic indices of radiotherapy. The two-year overall survival in this study was 30%, which is consistent with recent studies [1].

Flaxseed administration in the muffin formulation was not feasible due to a range of factors including patient anorexia, odynophagia and tolerability of the flaxseed muffin formulation. However, quantification of flaxseed bioavailability and urinary markers of systemic oxidative stress could be achieved. There was a trend towards decreased rates of pneumonitis in FS versus control (0% vs. 42.9%). Dietary flaxseed may be clinically useful as an agent to increase the therapeutic index of thoracic RT by increasing the radiation tolerance of organs at risk including the esophagus and lungs. Based on our preliminary data, further clinical trials investigating different formulations of flaxseed which are now available are warranted.

## Figures and Tables

**Table 1:** Patient and Tumor Characteristics

	*Control (n=7)*	*FS (n=7)*
Age, mean (range)	73.4 (57–81)	59.3 (35–79)
T stage		
T1	1 (14%)	1 (14%)
T2	3 (43)	2 (29)
T3	2 (29)	3 (43)
T4	1 (14)	1 (14)
N stage		
N1	1 (14)	1 (14)
N2	5 (71)	4 (57)
N3	1 (14)	2 (29)
M Stage		
M0	5 (71)	6 (86)
M1	2 (29)	1 (14)
Stage (AJCC 7th edition)		
IIIA	3 (43)	4 (57)
IIIB	2 (29)	2 (29)
IV	2 (29)	1 (14)
Location of primary tumor		
RUL	5 (71)	4 (57)
RML	0	1 (14)
RLL	0	1 (14)
LUL	1 (14)	0
LLL	1 (14)	1 (14)
Dose (cGy), median (range)	7200 (7000–8000)	7000 (5000–8000)
Histology		
squamous cell carcinoma	2 (29)	3 (43)
adenocarcinoma	3 (43)	2 (29)
sarcomatoid carcinoma	1 (14)	1 (14)
NSCLC, other	1 (14)	1 (14)
Chemotherapy		
Concurrent	1 (14)	3 (43)
Sequential	5 (71)	3 (43)
No chemotherapy	1 (14)	1 (14)
Chemotherapy Regimen		
Carboplatin and docetaxol	3 (43)	2 (29)
Carboplatin and pemetrexed	1 (14)	0
Carboplatin and gemcitabine	1 (14)	1 (14)
Cisplatin and docetaxol	0	2 (29)
Cisplatin and pemetrexed	1 (14)	1 (14)
Pulmonary Function, mean (range)		
FEV1 (L)	1.365 (1.29–1.44)	2.815 (0.88–4.07)
DLCO (%)	61.5 (43–80)	59.5 (39–97)
Tobacco History		
Current smoker	0	2 (29)
Former smoker	6 (86)	5 (71)
Pack-Years, median (range)	45 (20–120)	45 (3.5–100)
Non-smoker	1 (14)	0

**Table 2A:** Subject compliance and tolerability

	*Control (n=7)*	*FS (n=7)*	*p* value
Tolerability	5	3	0.59*
Reason for termination			
New metastatic disease	1	1	
Inability to tolerate muffin	1	3	
Muffin ingestion (% of prescribed)*, mean (range)*	73 (8.5–100)	37 (2.8–100)	0.23**
Blood and urine collection time points, *n*			
Pre-RT	2	4	
Week 1	2	4	
End RT	2	3	
1 mo post-RT	2	2	

* *P* value calculation by Fisher’s exact test (2-sided)

** *P* value calculation by Wilcoxon rank sum test

**Table 2B:** Esophagitis and pneumonitis rates in FS and control groups.

		*FS*	*Control*	p value*
Esophagitis	Grade 1–4	4	6	*0.56*
	Grade 0	3	1	
	Grade 1	1	2	
	Grade 2	3	4	
	Grade 3	0	0	
	Grade 4	0	0	
Pneumonitis	Grade 1–4	0	3	*0.19*
	Grade 0	7	4	
	Grade 1	0	0	
	Grade 2	0	3	
	Grade 3	0	0	
	Grade 4	0	0	

* P-value calculated by Fisher’s exact test (2-sided)
